# Detection of *Anaplasma Phagocytophilum* in Horses With Suspected Tick-Borne Disease in Northeastern United States by Metagenomic Sequencing

**DOI:** 10.3389/fvets.2021.673193

**Published:** 2021-06-09

**Authors:** Murugan Subbiah, Nagaraja Thirumalapura, David Thompson, Suresh V. Kuchipudi, Bhushan Jayarao, Deepanker Tewari

**Affiliations:** ^1^Pennsylvania Veterinary Laboratory, Harrisburg, PA, United States; ^2^Animal Diagnostic Laboratory, Pennsylvania State University, University Park, PA, United States; ^3^Center for Infectious Disease Dynamics, Pennsylvania State University, University Park, PA, United States

**Keywords:** tick-borne disease, anaplasma, next- generation sequencing, metagenoimcs, blood, microbiome, horse

## Abstract

Metagenomic sequencing of clinical diagnostic specimens has a potential for unbiased detection of infectious agents, diagnosis of polymicrobial infections and discovery of emerging pathogens. Herein, next generation sequencing (NGS)-based metagenomic approach was used to investigate the cause of illness in a subset of horses recruited for a tick-borne disease surveillance study during 2017–2019. Blood samples collected from 10 horses with suspected tick-borne infection and five apparently healthy horses were subjected to metagenomic analysis. Total genomic DNA extracted from the blood samples were enriched for microbial DNA and subjected to shotgun next generation sequencing using Nextera DNA Flex library preparation kit and V2 chemistry sequencing kit on the Illumina MiSeq sequencing platform. Overall, 0.4–0.6 million reads per sample were analyzed using Kraken metagenomic sequence classification program. The taxonomic classification of the reads indicated that bacterial genomes were overrepresented (0.5 to 1%) among the total microbial reads. Most of the bacterial reads (~91%) belonged to phyla Firmicutes, Proteobacteria, Bacteroidetes, Actinobacteria, Cyanobacteria and Tenericutes in both groups. Importantly, 10–42.5% of Alphaproteobacterial reads in 5 of 10 animals with suspected tick-borne infection were identified as *Anaplasma phagocytophilum*. Of the 5 animals positive for *A. phagocytophilum* sequence reads, four animals tested *A. phagocytophilum* positive by PCR. Two animals with suspected tick-borne infection and *A. phagocytophilum* positive by PCR were found negative for any tick-borne microbial reads by metagenomic analysis. The present study demonstrates the usefulness of the NGS-based metagenomic analysis approach for the detection of blood-borne microbes.

## Introduction

Tick-borne pathogens pose a growing threat to both animals and public health because ticks often harbor multiple known and unknown pathogens and geographic range of ticks is expanding in recent decades (1). Ticks are known to transmit bacteria, viruses and protozoal pathogens, and tick-borne pathogens account for much of vector-borne diseases in temperate regions of North America, Europe and Asia (2). Important tick-borne diseases of horses include Lyme disease, equine granulocytic anaplasmosis, Tick-borne Encephalitis Virus (TBEV) and equine Piroplasmosis (3–6). While Lyme disease and equine granulocytic anaplasmosis are frequently reported in horses in the United States, TBEV and equine piroplasmosis are considered non-endemic (3–6).

Diagnosis of tick-borne diseases can be challenging due to non-specific clinical signs and transmission of multiple pathogens by ticks (7). Diagnosis is commonly based on history of tick bite, clinical suspicion, serology, and detection of antigen or pathogen nucleic acid. Although serology is a primary method of diagnosis, it lacks sensitivity early during infection due to absence of detectable levels of antibodies and may also lack specificity due to cross-reactive antibodies (8). Furthermore, demonstration of pathogen-specific antibodies does not differentiate between current infection and past exposure. In contrast, PCR assays are highly sensitive and specific. However, use of single or multiplex PCR assays may result in missed detection of non-targeted or unknown etiologies and therefore strategies targeting multiple pathogens have been attempted with limited success (9). In this context, Next Generation Sequencing (NGS)-based metagenomic approach has a potential for the detection of diverse microbial pathogens and discovery of novel/unknown etiologies of infectious diseases (10–12). However, limited studies have examined the feasibility of using NGS-based metagenomic analysis for the diagnosis of tick-borne diseases in either humans or animals (13, 14).

In this study, we investigated use of metagenomic based NGS analysis of blood microbiome from horses with suspected tick-borne disease and compared it to apparently healthy horses. The study established the feasibility of NGS-based metagenomic shot gun approach for the detection of tick-borne pathogens in blood samples.

## Materials and Methods

### Blood Sample and Extraction of Total Genomic DNA

A subset of blood samples (*n* = 10) were randomly selected for metagenomic analysis from a larger cohort of horses suspected of having tick-borne diseases (TBD) recruited for studying prevalence of *Anaplasma phagocytophilum* and *Borrelia burgdorferi* infections during 2017–2019. In addition, blood samples (*n* = 5) from apparently healthy horses that were not part of the TBD study cohort were included in the metagenomic study. The horses with suspected tick-borne illnesses often had history of tick exposure and showed clinical signs such as fever, depression, petechiae, and inappetence. The reported clinical signs among the horses included in the study are listed in Table 1 and horses suspected with tick-borne infection with clinical signs are referred as “sick group.”

**Table 1:** Demographic, clinical and laboratory analyses for horses included in the study.

Sample ID^*^	**Age (years)**	**Temperature (^**°**^C)**	**Metagenome analysis**	**PCR**	**Presence of morulae**	**Clinical parameters and history**
SAMN18751441	3	39.66	0	0	No	Inappetence, Tick exposure.
SAMN18751440	21	38.94	1	1	Yes	Depression; edema.
SAMN18751442	18	40.33	1	1	Yes	Depression, petechiae and inappetence, low hematocrit values.
SAMN18751435	25	40	0	0	No	Depression, petechiae, inappetence.
SAMN18751436	21	40	0	0	No	Inappetence.
SAMN18751437	22	40.33	1	1	No	Depression, inappetence, tick exposure, edema and petechiae.
SAMN18751439	NA	40.56	0	1	No	Depression and tick exposure.
SAMN18751438	7	NA	1	1	Yes	Low hematocrit values.
SAMN18751447	18	40.56	1	0	No	Depression, petechiae and inappetence.
SAMN18751443	1	NA	0	1	No	None reported.
SAMN18751444	17	37.22	0	0	No	Healthy
SAMN18751445	15	37.22	0	0	No	Healthy
SAMN18751446	9	37.22	0	0	No	Healthy
SAMN18751448	22	37.22	0	0	No	Healthy
SAMN18751449	25	37.22	0	0	No	Healthy

* *The metagenome data can be accessed at https://www.ncbi.nlm.nih.gov/sra/PRJNA722464*.

*NA, not available; PCR and Next-Generation Metagenomic analyses: 0 - negative and 1 - positive*.

Blood samples were collected in EDTA vacutainer tubes (BD Bioscience, San Jose, CA). The total genomic DNA from blood samples were extracted using the Blood or Body Fluids Spin Protocol (QIAmp DNA extraction mini kit, Qiagen, Germantown, MD) following the manufacturer's instructions. The extracted DNA was not treated with RNAase and the quality and quantity of the DNA were analyzed by spectrophotometry (Nanodrop, Thermo Fisher Scientific, Waltham, MA) and fluorometric method (Qubit, Thermo Fisher Scientific, Waltham, MA), respectively.

### Microbial DNA Enrichment

Microbial DNA from the horse blood samples was enriched using the Illustra DNA enrichment kit (Biolabs, New England, MA). Briefly, up to 1 μg of total nucleic acid extracted from blood samples was subjected to illustra DNA enrichment protocol, which binds and removes a proportion of mammalian genomic DNA. These enriched DNA were then amplified using Genomiphi DNA amplification kit (GE Healthcare, UK) and the quantity of enriched and amplified blood DNA was estimated using Qubit.

### Sequencing

Between 100 to 500 ng of enriched and amplified genomic DNA samples were used to prepare library for NGS using Nextera DNA Flex Library Preparation Kit (Illumina, San Diego, CA) according to the manufacturer's protocol. The quantity and the fragment size of libraries were measured using Qubit and Bioanalyzer (Agilent, Santa Clara, CA), respectively. The individual libraries were normalized according to the manufacturer's protocol and then pooled (5–6 samples per pool) before loading into MiSeq instrument (Illumina, San Diego, CA). MiSeq Reagent Kit v2 (2 × 150; 2 × 250 cycles) was used for sequencing the DNA libraries.

### Bioinformatics Analysis

The MiSeq run quality was checked using Illumina Sequencing Analysis Viewer. The trimmed reads from MiSeq runs were collected as fastaq files, analyzed in cloud based BaseSpace (Illumina) platform and the quality was analyzed using Quast application (quast.sourceforge.net). Samples that contained good quality reads (Quast default standards) were further analyzed for the taxonomic identification using Kraken metagenomic analysis application v2.0.1 that uses an exact-alignment database queries of k-mers from each read (15). A subset of reads categorized as unidentified by Kraken were mapped to determine the taxa using nucleotide blast search in NCBI. The detection threshold for microbial DNA reads was set at 1% of total microbial reads and microbial phylum/families/genera/species reads that constitute > 1% of the respective microbial taxonomy were considered for further analyses. The Shannon indices for richness and diversity and Simpson's index for evenness of microbial families/genera/species (16) were estimated using online statistical tools (datanalytics.org.uk; easycalculation.com). Hutchinson's *t*-test was used to estimate the statistical significance of diversity index between healthy and sick groups. Average percent of each microbial family/genus/species were estimated for both healthy and sick groups and the prominent microbial families/genera/species that are relevant to equine infections are discussed more in details.

#### Anaplasma Phagocytophilum Real-Time PCR

*A. phagocytophilum msp2* gene was amplified and detected using a real-time PCR as described previously (17).

## Results

### NGS Data Analysis and Quality Control

A total of 8,826,219 good quality reads were generated with an average of 0.4–0.6 million reads per sample from apparently healthy horses (*n* = 5) and horses with suspected tick-borne infection (*n* = 10). The sequence data of this project were submitted to NCBI, BioProject reference number PRJNA722464 (https://www.ncbi.nlm.nih.gov/sra/PRJNA722464). Overall, 95–97% of the reads from each sample were considered belonging to host DNA. Microbial reads contributed to 1.1 and 1.3% of total reads from healthy and sick samples, respectively (Table 2). The remaining non-host genome reads were mapped to microbes from other Eukarya and plant kingdom (data not shown). Approximately 97 to 98% of the microbial reads were assigned to bacteria, virus, fungi and apicomplexan groups. Overall, no differences were noted in the percentages of total microbial and host reads between these two groups of animals.

**Table 2:** The composition (%) of major circulating microbial DNA present in healthy and sick equine blood samples.

	**Healthy (*n* = 5)**	**Sick (*n* = 10)**
Bacteria	0.80	0.72 ± 0.06 (0.5–1.0)
Virus	0.01 ± 0.004 (0.01–0.03)	0.01
Fungi	0.16 ± 0.02 (0.1–0.2)	0.29 ± 0.13 (0.11–1.5)
Apicomplexa	0.14 ± 0.02 (0.1–0.16)	0.24 ± 0.12 (0.1–1.3)
Archaea	0.03	0.02
# of reads^*^	386,375 ±113,142 (139,595–753,414)	653,318 ± 188,828
		(80,491–1,965,941)

*The percentages and standard errors were estimated out of total number of reads (for fungi and apicomplexa out of total number of domain eukaryote reads). The values in the parenthesis indicate the range; n = number of samples*.

* *The mean number of reads with standard error of mean and the range are included*.

### Microbial Content of Blood Samples

Analysis of microbial reads using Shannon and Simpson diversity indices showed presence of a diverse population of microbial DNA comprising apicomplexan parasites, bacteria, viruses and fungi in both healthy and sick horses (*P* > 0.01). Notably, the low (below 0.5) Simpson's evenness index was suggestive of uneven distribution of the microbial genera in both sick and healthy horses (Supplementary Table 1).

### Bacterial Diversity in Equine Blood

**Figure 1:**
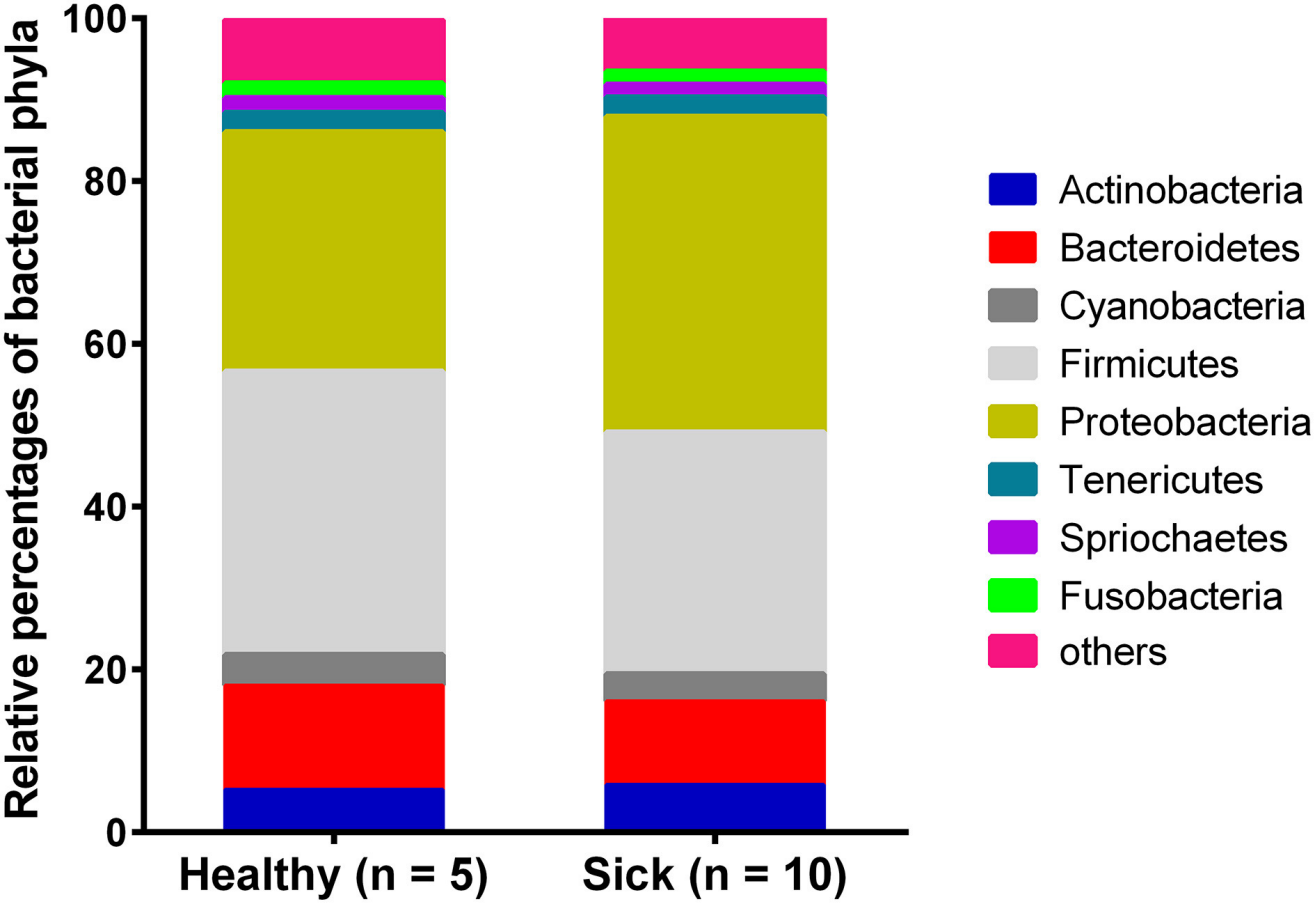
Relative abundance of circulating bacterial phyla reads from horses with suspected tick-borne infection (sick) and apparently healthy animals. The percentages were estimated out of total bacterial reads.

Most of the bacterial reads (~91%) from horses with suspected tick-borne infection and apparently healthy group were assigned to the phyla belonging to Firmicutes, Proteobacteria, Bacteroidetes, Actinobacteria, Cyanobacteria and Tenericutes (Figure 1). Bacterial Phyla that represented <2% of total bacterial reads were not included for analysis. Notably, a higher level of Proteobacteria was found in horses with suspected tick-borne infection compared to apparently healthy horses. In contrast, a higher proportion of Bacteroidetes and Firmicutes genomes were found in apparently healthy horses compared to sick horses.

**Figure 2:**
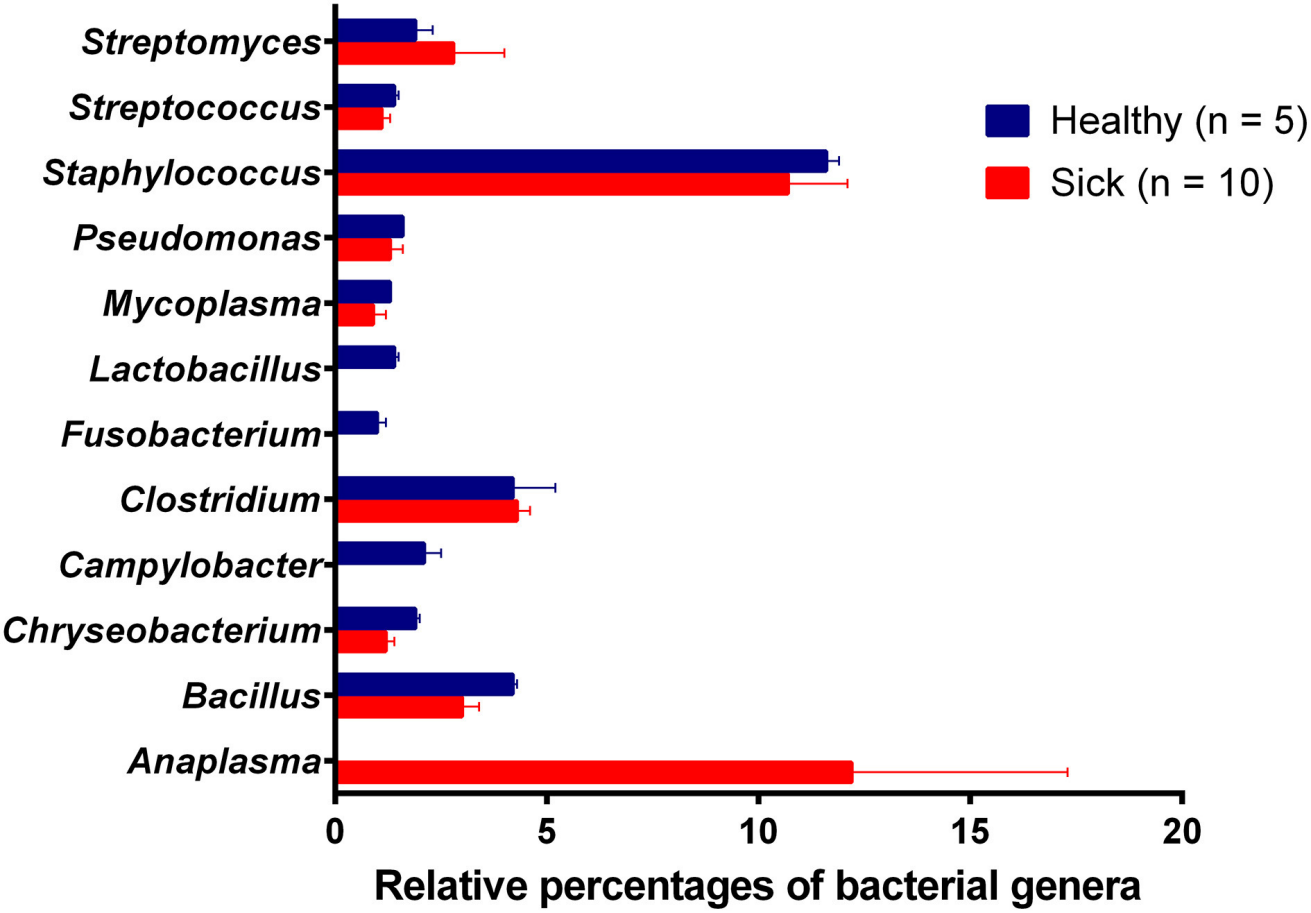
Relative abundance (average percentage + standard error) of bacterial genera. No difference (*P*-value = 0.54) in the diversity of bacterial genera between healthy (H = 2.28) and sick (H = 2.18) groups. Simpson's evenness scores show uneven distribution of bacterial species in both apparently healthy (0.42) and sick (0.33) groups.

Analysis of bacterial reads at genus level showed a diverse composition [Shannon diversity index (H) > 2 for both apparently healthy and sick groups, Supplementary Table 1] of microbial population comprising multiple genera (Figure 2). *Anaplasma* was the most abundant genus in 5 of the 10 samples (range 10–42.5%) from sick horses and was only present in the samples from sick horses. Notably, four out of five samples containing *Anaplasma phagocytophilum* reads by metagenomic analysis were also positive by a *A. phagocytophilum* PCR. One animal from the sick group that was positive for *A. phagocytophilum* reads by metagenomic analysis tested negative by PCR. Two animals from the sick group that tested positive for *A. phagocytophilum* by PCR did not have any *Anaplasma* sp. reads by metagenomic analysis (Table 1). Genera *Bacillus* and *Chryseobacterium* were relatively more abundant in apparently healthy horses and reads for genera *Campylobacter, Fusobacterium*, and *Lactobacillus* were found exclusively in healthy horses (Figure 2). Overall, *A. phagocytophilum* was the most abundant bacterial species found in animals with suspected tick-borne infection (Table 3).

**Table 3:** Major bacterial species detected with equine blood metagenomic analysis.

**Bacterial species**	**Percentage of bacterial reads**	
	**Healthy (*n* = 5)**	**Sick (*n* = 10)**
Anaplasma phagocytophilum	0	12.2 ± 5.1
Staphylococcus aureus	9.0 ± 0.4	7.9 ± 1.7
Staphylococcus simulans	0	1.4 ± 0.7
Streptomyces sp. ICC1	0	1.4 ± 0.9

*The percentages and standard error were estimated out of total number of bacterial reads. n = number of samples*.

### Distribution of Viruses, Fungi and Apicomplexa

The viral reads represented a very small proportion, ~0.01% of total reads analyzed (Table 2), belonging mainly to DNA viruses. Even with very small percentages of reads, no significant differences were observed in the major viral families between the two groups of horses. Similarly, no significant differences were observed in the percentages of fungal or Apicomplexa reads between the sick and healthy groups. These findings were considered of no significance due to low read counts.

## Discussion

Recent advances in Next Generation Sequencing (NGS)-based metagenomic analysis have potential clinical applications including diagnosis of infectious diseases, host microbiome analysis, host immune response analysis and oncology (10, 11). NGS-based metagenomic analysis can involve either targeted amplicon sequencing such as 16S ribosomal RNA genes or shotgun sequencing. Shotgun sequencing allows unbiased analysis of partial or complete microbial genomes, transcriptomes and viromes from diagnostic specimens. However, NGS-based metagenomic shotgun sequencing is more expensive, requires greater sequencing depth for the detection of rare or less abundant targets and generates greater amounts of data that requires advanced computational tools for storage and bioinformatic analysis compared to targeted sequencing (11). Metagenomic approach for infectious diseases diagnostics has the potential for diagnosis of mixed infections, detection of associated virulence and antimicrobial resistance genes and discovery of novel/unknown etiologies (18).

In the present study, we used metagenomic based shotgun NGS for analysis of blood microbiome in sick horses with clinical suspicion of tick-borne disease and compared it with the blood microbiome of apparently healthy horses. The proportion of microbial (~1.1%) reads, especially bacterial reads (0.8%) detected out of total genomic reads in horse blood were comparable to those found in the blood of humans with acute leukemia (19). A key observation of our study is that while the proportion of the microbial DNA was comparable between healthy and sick horses, there were differences in the composition of the microbial DNA in blood between these two groups.

The higher proportion of phyla proteobacteria detected in horses with suspected tick-borne infection compared to apparently healthy group directly correlated with the detection of *Anaplasma phagocytophilum* reads in the former group. This finding was further confirmed by a PCR targeted to detect *A. phagocytophilum* and also correlated with the history of tick-bite and with one or more clinical signs including fever, anorexia, depression, petechial hemorrhage on conjunctival membrane and icterus or detection of morulae in blood smears. Genus *Anaplasma* is classified under *Alphaproteobacteria*, and *Anaplasma phagocytophilum* is an important tick-borne bacterium causing disease in horses (20). Currently, PCR for the detection of *A. phagocytophilum* DNA in blood and demonstration of a 4-fold or greater increase in the antibody titer by an Indirect Fluorescent Antibody (IFA) test are commonly employed for diagnosis of equine granulocytic anaplasmosis (5). The finding of one blood sample from the sick group showing presence of *A. phagocytophilum* by metagenomic analysis but negative by PCR was possibly due to PCR inhibition. The two samples from the sick group that were positive by PCR but negative by metagenomic analysis was likely due to lower sensitivity of NGS metagenomic method compared to amplification-based assays (21).

Non-detection of any other significant pathogen by metagenomic analysis is likely due to small sample size and sampling bias as the sick animals included in the study were selected based on high index of suspicion for tick-borne diseases. It is worth noting that *A. phagocytophilum* and *Borrelia burgdorferi* are prevalent tick-borne pathogens in the region where the study was conducted (3, 5). Rarity of detection of *B. burgdorferi* in blood of infected animals may explain non-detection of *B. burgdorferi* reads by metagenomic analysis (22, 23). Our results suggest that sequencing of microbial DNA from blood using NGS can be used as a diagnostic tool for unbiased detection of blood-borne pathogens and is consistent with a previous study that reported detection of vector-borne pathogens in five out of eight known positive human blood samples using metagenomic shotgun sequencing method (13). Recent studies have demonstrated utility of targeted amplicon NGS metagenomic approach for the detection vector-borne bacteria and protozoan haemoparasites in canine blood samples (24, 25).

The distribution of viral, fungal and apicomplexan families/genera/species did not differ significantly among healthy and sick horses and significance of these findings is uncertain due to low read counts. Blood has traditionally been considered devoid of microbes in healthy individuals. However, recent metagenomic studies provide evidence for the presence of signature fragments of bacterial, viral and other microbial nucleic acids in blood of healthy human beings (26). Presence of phyla including Proteobacteria, Actinobacteria, Firmicutes and Bacteroidetes in blood of healthy human beings has also been reported based on analysis of DNA and RNA (27). In addition, some studies demonstrated the presence of viable bacteria in blood of healthy human beings (28).

A major advantage of sequencing is that the method can identify mutant and variant microbes (strain level identification), particularly important for viruses, that PCR assays might fail to identify. Furthermore, sequencing can also provide valuable insights for studying pathogen evolution, which is not possible with PCR-based methods. However, application of NGS metagenomics in microbial diagnostics is still limited due to high cost, complexity of data, potential for contamination, difficulty in discerning clinical relevance of sequencing data and need for standardization of NGS methods for diagnostic applications (18, 29).

NGS based testing is still an evolving field and some of the challenges we encountered during this study were choosing an appropriate platform for the analyses of the millions of sequenced reads, setting a threshold value to parse the lowest abundant taxa, deciding the appropriate enrichment strategy of microbial genomes and exclusion of host DNA. In the current study we used Kraken metagenomics analysis application from Basespace (Illumina Inc., San Diego, CA). The Kraken metagenomics analysis uses long exact sequence matches alignment when classifying short-read sequences and label almost all the reads sequenced that has exact matches unlike programs which label sequences that are most abundant (15). These qualities of chosen analytical platform may be essential to identify the lowest abundant microbial taxa. In this study we used 1% of specific reads out of total respective microbial reads as a threshold for a phylum/genus/species to be included in the analysis. We recognize that there is a potential risk of excluding low abundant but important microbial reads with such a strategy (29). Lastly, our study focus was on establishing a methodology with a small sample set. Expanding the study to a large population and including variety of disease conditions besides tick borne illness would be valuable.

Overall, the findings of metagenomics analysis of blood samples from apparently healthy horses and horses with suspected tick-borne infection suggest that the approach can be used to detect blood borne pathogens. Additional studies to establish a baseline of potential circulating background microbial DNA in healthy animals would be useful to parse the non-significant microbial sequences.

## Data Availability Statement

The original contributions presented in the study are publicly available. This data can be found here: NCBI repository, BioProject ID: PRJNA722464.

## Ethics Statement

Ethical review and approval was not required for the animal study because the study involves a specimen set that were already collected as part of State survey for monitoring animal diseases for disease investigation authorized in the State of Pennsylvania. A subset of stored samples from this survey meeting qualifications were investigated in this study at an official State veterinary diagnostic facility.

## Author Contributions

MS carried out the study, analyzed data, and wrote manuscript. NT managed the study and wrote the manuscript. DTh helped in selecting samples. SK, BJ, and DTe coordinated and conceptualized the study and reviewed manuscript. All authors contributed to the article and approved the submitted version.

## Conflict of Interest

The authors declare that the research was conducted in the absence of any commercial or financial relationships that could be construed as a potential conflict of interest.
