# Supplementary material for: Detection of Anaplasma Phagocytophilum in Horses With Suspected Tick-Borne Disease in Northeastern United States by Metagenomic Sequencing
Source: Front Vet Sci. 2021 Jun 9;8:673193. doi: 10.3389/fvets.2021.673193 (PMC8219919; doi:10.3389/fvets.2021.673193)
Supplement: Supplementary file 1 [file Table_1.DOCX]

**Supplementary Material**

**Supplementary Table 1.** Shannon and Simpson values for diversity and evenness of microbial reads in blood of sick and healthy horses obtained by metagenomic Next-generation sequencing.

|  | **Apicomplexa** | | | **Bacteria** | | | | | | **Virus** | | | | | |
| --- | --- | --- | --- | --- | --- | --- | --- | --- | --- | --- | --- | --- | --- | --- | --- |
|  | **Genera** | | | **Genera** | | | **Phyla** | | | **Genera** | | | **Family** | | |
|  | Healthy | | Sick | Healthy | | Sick | Healthy | | Sick | Healthy | | Sick | Healthy | | Sick |
| Shannon Diversity Index (H) | 0.92 | | 0.91 | 2.28 | | 2.18 | 1.41 | | 1.38 | 2.3 | | 2.1 | 1.6 | | 2.3 |
| Hutchinson’s t-test: t-stat, df | 0.1; 139 | | | 0.5; 57 | | | 0.89; 908 | | | 1.6; 390 | | | 1.1; 716 | | |
| Hutchinson’s t-test: CI | 0.1 | 0.23 | | 0.15 | 0.38 | | 0.07 | 0.05 | | 0.14 | 0.15 | | 0.07 | 0.01 | |
| Hutchinson's t-test, *P* value | *0.75* | | | *0.61* | | | *0.38* | | | *0.15* | | | *0.07* | | |
| Simpson's Index (D) | 0.59 | | 0.59 | 0.15 | | 0.15 | 0.29 | | 0.31 | 0.18 | | 0.2 | 0.13 | | 0.15 |
| Simpson's Diversity Index | 0.41 | | 0.41 | 0.85 | | 0.85 | 0.71 | | 0.69 | 0.82 | | 0.7 | 0.87 | | 0.86 |
| Simpson's Evenness | 0.28 | | 0.28 | 0.42 | | 0.33 | 0.56 | | 0.56 | 0.33 | | 0.8 | 0.41 | | 0.77 |

**Shannon diversity index (H)**: Measures the diversity of the microbial composition at family/genera/species level. Diversity index value towards 1 and higher indicates higher richness and evenness.

**Simpson’s diversity index**: Measures the probability that two individuals randomly selected from a sample will belong to different family/genera/species. The value ranges between 0 and 1, the greater the value the greater the diversity**.**

**Simpson’s evenness:** Show the relative abundance of individual family/genera/species in a community. Score ranges between 0 and 1 with score towards 0 suggests less evenness i.e., some family/genera/species are more abundant compared to other.

t-stat – t-statistics; df – degrees of freedom; CI – confidence interval
